# The clinical significance of spondin 2 eccentric expression in peripheral blood mononuclear cells in bronchial asthma

**DOI:** 10.1002/jcla.23764

**Published:** 2021-05-16

**Authors:** Peng Zhou, Cai‐xia Xiang, Jin‐feng Wei

**Affiliations:** ^1^ Department of Pediatric Shengzhou People’s Hospital (the First Affiliated Hospital of Zhejiang University Shengzhou Branch) Shengzhou China; ^2^ Department of Respiratory Hangzhou Children’s Hospital Hangzhou China

**Keywords:** bronchial asthma, diagnosis, GO analysis, peripheral blood mononuclear cell, spondin 2

## Abstract

**Background:**

Bronchial asthma (BA) was a heterogeneous disease characterized by chronic airway inflammation. Spondin 2 (SPON2) was reported to be implicated in the integrin pathway, protein metabolism, and drug‐induced lupus erythematosus. The purpose of this study was to evaluate the significance of SPON2 in BA diagnosis and treatment.

**Methods:**

Peripheral blood samples were obtained from 137 BA pediatric patients (61 mild‐to‐moderate BA and 76 severe BA) and 59 healthy children. Subject's information, clinical indexes, pulmonary ventilation functions were recorded in the two groups. Peripheral blood mononuclear cells (PBMCs) were isolated from patients’ samples. qRT‐PCR and ELISA assays were employed to examine the levels of SPON2 and inflammatory cytokines, respectively. Pearson's correlation analysis confirmed the association between SPON2 and inflammatory cytokines. Receiver operating characteristic (ROC) analysis was used to evaluate the potentials of SPON2 in terms of BA detection and discriminating against the severity of BA.

**Results:**

Bioinformatics analysis showed that SPON2, OLFM4, XIST, and TSIX were significantly upregulated, while KDM5D and RPS4Y1 were reduced in BA. GO analysis verified that these six genes were mainly involved in neutrophil degranulation, neutrophil activation involved in immune response, neutrophil activation, and neutrophil‐mediated immunity. After isolating PBMCs, we found that SPON2 was remarkably increased in BA pediatric group compared with healthy children, and the relative levels of SPON2 were related to the severity of BA. The receiver operating characteristic (ROC) analysis revealed the high potentials of SPON2 in BA diagnosis (AUC was 0.8080) and severity distinctions (AUCs were 0.7341 and 0.8541, respectively). Also, we found that there were significant differences in fractional exhaled nitric oxide (FeNO), forced expiratory volume in 1 s (FEV1)%, FEV1/ forced vital capacity (FVC)%, immunoglobulin E (IgE), serum eosinophils, and serum neutrophils between mild‐to‐moderate BA group and severe BA group. Finally, SPON2 was negatively correlated with IL‐12 while positively associated with IL‐4, IL‐13, and IL‐17A.

**Conclusions:**

SPON2 was a viable biomarker for diagnosing and degree of severity in BA, providing more insight into exploring BA and treatment's pathogenesis.

## Graphical abstract

Bioinformatic analysis showed that SPON2, OLFM4, XIST, and TSIX were significantly upregulated, while KDM5D and RPS4Y1 were reduced in BA. GO analysis verified that these six genes were mainly involved in neutrophil degranulation, neutrophil activation involved in immune response, neutrophil activation, and neutrophil‐mediated immunity. After isolating PBMCs, we found that SPON2 was remarkably increased in BA pediatric group compared with healthy children, and the relative levels of SPON2 were related to the severity of BA. The receiver operating characteristic (ROC) analysis revealed the high potentials of SPON2 in BA diagnosis (AUC was 0.8080) and severity distinctions (AUCs were 0.7341 and 0.8541, respectively). Also, we found that there were significant differences in fractional exhaled nitric oxide (FeNO), forced expiratory volume in 1s (FEV1)%, FEV1/ forced vital capacity (FVC)%, immunoglobulin E (IgE), serum eosinophils, and serum neutrophils between mild‐to‐moderate BA group and severe BA group. Finally, SPON2 was negatively correlated with IL‐12 while positively associated with IL‐4, IL‐13, and IL‐17A.

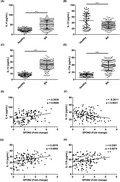

## 1 INTRODUCTION

Bronchial asthma (BA) was a heterogeneous chronic respiratory disease,^1^ characterized by airway inflammation,^2^ associated with variable airflow obstruction, airway hyperresponsiveness, and airway wall remodeling.^3^ Some studies showed that BA was deemed a multifactor disease affected by genetic and environmental factors,^4^ with clinical manifestations, including recurrent chest tightness, wheezing phlegm, dyspnea, or cough.^5^, ^6^ However, its exact mechanism remained mostly uncertain. Hence, BA is mostly hard to diagnose. BA threatened the health of 4.3% of the world's population, which was still on the rise and increased the burden on families and health care systems.^8^ More importantly, the health of children with BA was inevitably affected as well.^9^ To date, the bronchial provocation test was a standard method to check airway hyperresponsiveness.^10^ Due to safety considerations and the lack of reliable BA detection methods, the clinical application of children excitation tests was severely limited.^11^ Therefore, it was imperative to find novel biomarkers to detect and treat BA, thereby contributing to early intervention and BA treatment.

Eosinophils (EOS) were recognized in 1879 by Paul Ehrlich.^12^ However, for 100 years, the role of EOS in BA remained unknown. At the beginning of the 21st century, EOS was poorly found in peripheral blood and lung tissues in healthy people.^13^ Meanwhile, in 1975, Horn et al^14^ found that most BA patients’ severity was positively correlated with increased EC levels in blood sputum, as did their airway responsiveness. Subsequently, some scholars also verified that once BA patients inhaled allergens, EOS levels were prominently increased in bronchoalveolar lavage fluid (BALF) and lung tissues.^15^, ^16^ BA was considered a chronic inflammatory disease dominated by mast cells (MC) and EOS infiltration.^17^ According to a previous study, spondin 2 (SPON2) was confirmed to be associated with EOS.^18^ SPON2, a cell adhesion protein, facilitated adhesion, and outgrowth of embryonic neurons.^19^, ^20^ Through GO analysis, SPON2 was found to be involved in the initiation of the innate immune response,^21^positive regulation of interleukin‐6 (IL‐6), macrophage cytokine, and tumor necrosis factor production.^22^, ^23^ These studies above suggested the potential of SPON2 in BA. Here, in this study, we detected the role of SPON2 in detecting pediatric BA patients from healthy children and its clinical application in treatment.

## 2 METHODS

### 2.1 Bioinformatics analysis

The original differentially expressed genes used in this study were downloaded from the National Center for Biotechnology Information Gene Expression Omnibus (NCBI‐GEO; https://www.ncbi.nlm.nih.gov/geo/). Gene ontology (GO) enrichment analysis was implemented using DAVID (http://david.abcc.ncifcrf.gov/). GO terms with values less than 0.05 (molecular functions, biological processes, and cellular components) were considered to be significantly enriched for differentially expressed genes. The Kyoto Encyclopedia of Genes and Genomes (KEGG) was a database resource for understanding the high‐level functions and effects of biological systems (http://www.genome.jp/kegg/).

### 2.2 Subjects recruitment

A total of 137 pediatric BA patients and 59 healthy children who underwent physical examination were enrolled between December 2016 and April 2019 at Hangzhou Children's Hospital. All BA children were divided into a mild‐to‐moderate group (61 cases) and a severe group (76 cases). According to the Global Initiative for Asthma (GINA) guidelines released in 2015 and the Asthma Control Test (ACT) score, BA patients were diagnosed. The severity of BA was established and evaluated by FEV1% levels: mild‐to‐moderate BA (FEV1%, 60~80%) and severe BA (FEB1%, <60%). Exclusion criteria included: severe heart, liver, kidney, and lung diseases; patients who had a history of respiratory or chronic diseases; patients who used concomitant anti‐BA, anti‐allergic drugs, immunosuppressants, immunomodulators, or inflammatory transmitter antagonists at least 3 months before this study.

All participants’ parents or guardians signed the informed consent and completed a questionnaire concerning demographic information. The ethics approval of this study was obtained from the Ethics Committee of Hangzhou Children's Hospital. This study obeyed the Declaration of Helsinki in 1983.

### 2.3 Sample collection and PBMCs examination

3~5 ml fasting venous blood (heparin for anticoagulation) was taken from each child, and the plasma was separated below −30°C. The children in the onset stage were collected within 1~3 days of BA onset or before the use of hormone therapy, and the sample collection in the stable stage was at least 3 months from the last blood collection; there was no asthma onset in the last 6 weeks. 10 ml anticoagulant‐treated samples were placed in sterile centrifuge tubes, centrifuged at 4°C, 100 rpm for 5 min. The procedures of PBMCs extraction were described as follows: the anticoagulant‐treated samples were centrifuged at 4°C, 3000 rpm for 10 min; PBS was added to the anticoagulant tube and mixed well with a 3 ml dropper; two 15 ml centrifuge tubes were taken, and 3 ml lymphocyte separation solution was added, respectively; in each centrifuge tube, the lymphocyte separation layer was added with PBS, and the samples were thoroughly mixed; the mixed samples were centrifuged at 400 g for 30 min at room temperature; the extracted PBMCs were washed twice with Hanks’ solution and stored under 4°C until further analysis.

### 2.4 Lung function assessment and IgE level measurement

Lung function assessment was evaluated through a portable pediatric spirometer; this evaluation has been done in triplicate to get the best value. IgE levels were analyzed using a human ELISA IgE kit following the manufacturer's protocol.

### 2.5 Quantitative real‐time polymerase chain reaction assay (qRT‐PCR)

Total RNA was extracted from PBMCs using TRIzol reagent (Invitrogen) and quantified by spectrophotometer (absorbance at 260 nm and 280 nm). Then, cDNA was transcribed and synthesized using the cDNA Synthesis Kit (Vazyme) following the manufacturer's instructions. Afterward, qRT‐PCR was conducted using SYBR Green Assay Kit (Vazyme) on an ABI 7500 system. The thermal cycling conditions were as follows: initial denaturation at 94°C for 5 min, followed by 40 cycles of denaturation 94°C for 10 s, and annealing at 72°C for 10 s. The expression of SPON2 was normalized to housekeeping gene GAPDH and calculated according to the 2^−△△CT^ method. The primers were designed and synthesized by GenePharma. The primers sequences were as follows: SPON2 forward, 5’‐AAGAACCAGTACGTCAGTATCGG‐3’ and reverse, 5’‐CACAAACGAGACCAGCGAGT‐3’; OLFM4 forward, 5’‐GACCAAGCTGAAAGAGTGTGAGG‐3’ and reverse, 5’‐CCTCTCCAGTTGAGCTGAACCA‐3’; XIST forward, 5’‐ CTTAAAGCGCTGCAATTCGCT‐3’ and reverse, 5’‐AGGGTGTTGGGGGACTAGAA‐3’; TSIX forward, 5’‐TAGGCGTCCCATGAATAATAAAG‐3’ and reverse, 5’‐TCTCTAGCATCCCCACAAAAAT‐3’; KDM5D forward, 5’‐CAAGACCCGCTTG‐ GCTACATT‐3’ and reverse, 5’‐TTGGACGCGAGGAGTAAATCT‐3’; RPS4Y1 forward, 5’‐ATCCGCTACCCAGATCCTGT‐3’ and reverse, 5’‐GGCTCCACCAATCACCATAC‐3’; GAPDH forward, 5’‐TGCACCACAACTGCTTAGC‐3’ and reverse, 5’‐GGCATGGACTGTGGTCATGAG‐3’.

### 2.6 ELISA assay

IL‐4, IL‐12, IL‐13, and IL‐17A levels in plasma samples were determined by human IL‐4, IL‐12, IL‐13, and IL‐17A ELISA commercial kits following the manufacturer's instructions. ELISA commercial kits were bought from Abcam (Solarbio Life Science), and the detailed information was as follows: human IL‐4 ELISA kit (SEKH‐0011), human IL‐12 ELISA kit (SEKH‐0020), human IL‐13 ELISA kit (SEKH‐0022), and human IL‐17A ELISA kit (SEKH‐0026‐96T).

### 2.7 Statistical analysis

SPSS version 21.0 software was used in our study for data analysis. All data were presented as mean ± standard deviation. Student's *t* test, followed by Newman‐Keuls post hoc test, was used to distinguish between two groups. The results of the area under the curves (AUC) measured by receiver operating characteristic analysis were performed to evaluate the diagnostic value of SPON2, while Pearson's analysis was employed to verify the correlation between two factors. Statistical significance was set when a *p* value was less than 0.05.

## 3 RESULTS

### 3.1 Functions of differentially expressed genes in BA

**FIGURE 1:**
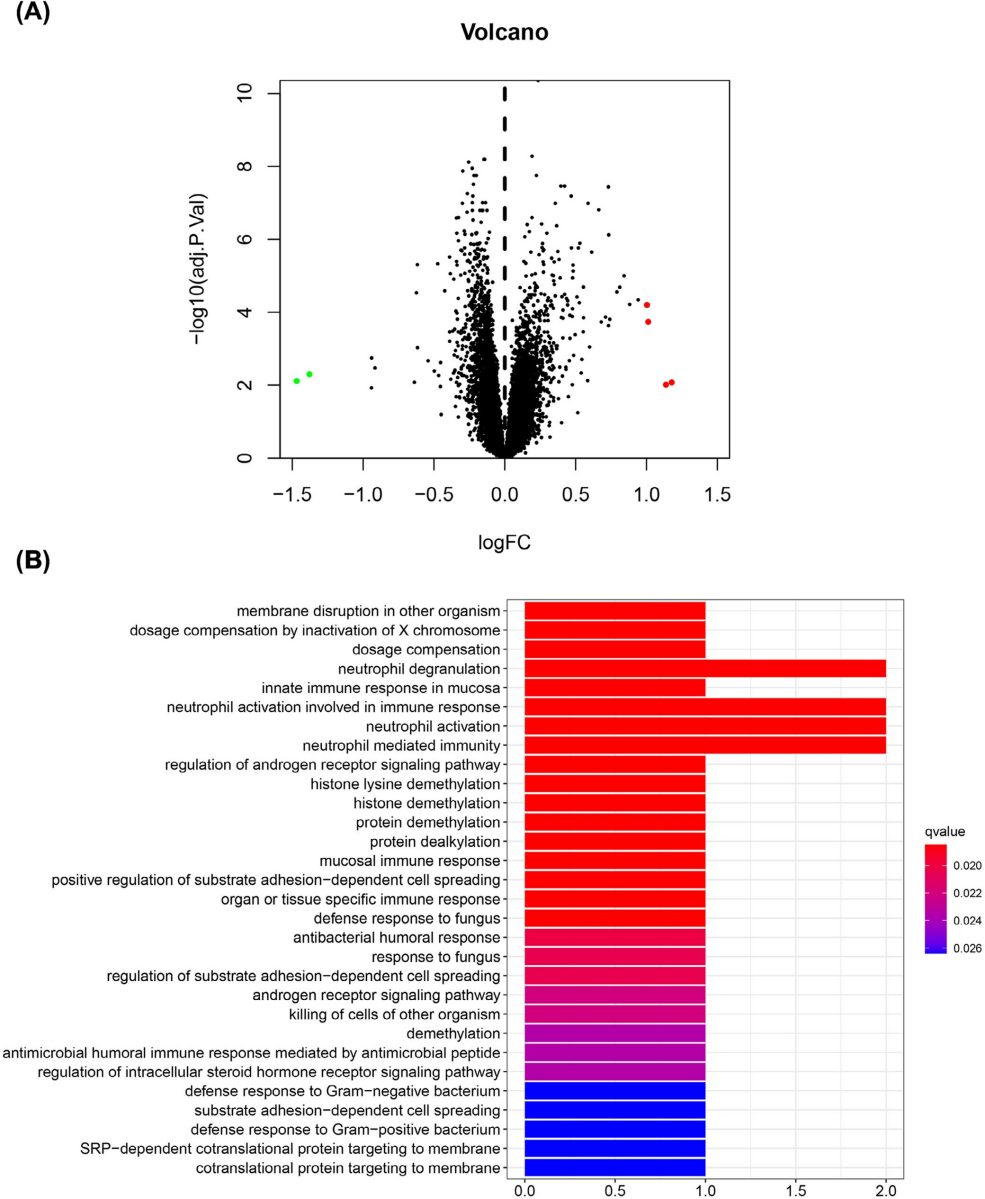
The functions of differentially expressed genes in BA. (A) Volcano plot of differentially expressed genes in BA. *x*‐axis: log_2_(fold change), *y*‐axis: ‐log_10_(adjusted *p* value). (B) GO analysis of differentially expressed genes in BA, including biological processes, cellular components, and molecular functions. BA, bronchial asthma

Using bioinformatics analysis, we identified six abnormally expressed genes in BA (shown in Table 1). Among them, SPON2, OLFM4, XIST, and TSIX were increased while RPS4Y1 and KDM5D were decreased (Figure 1A). Moreover, the GO analysis in Figure 1B further demonstrated that these genes were mainly implicated in neutrophil degranulation, neutrophil activation involved in immune response, neutrophil activation, and neutrophil‐mediated immunity.

**TABLE 1:** Differentially expressed genes in BA

Gene ID	LogFC	AveExpr	t	*p*	Adj. *p*	B
SPON2	1.0032	7.7948	4.9976	<0.0001	<0.0001	5.4905
OLFM4	1.0121	6.3387	4.6817	<0.0001	0.0002	4.0751
KDM5D	−1.3786	5.6721	−3.5983	0.0004	0.0050	−0.1285
RPS4Y1	−1.4688	7.3983	−3.4393	0.0006	0.0077	−0.6581
XIST	1.1778	6.1387	3.4037	0.0007	0.0084	−0.7738
TSIX	1.1373	6.4683	3.3506	0.0009	0.0097	−0.9438

### 3.2 Clinical characteristics of all subjects

A total of 196 subjects, including 59 healthy children who underwent a physical examination and 137 pediatric BA patients, has been employed in the present study. The clinical information of 196 subjects was displayed in Table 2. There were no differences in age, gender, and allergic family history between the two groups. Moreover, we divided BA patients into mild‐to‐moderate BA group and severe BA group. From Table 3, we found that there were significant differences in fractional exhaled nitric oxide (FeNO), forced expiratory volume in 1 s (FEV1)%, FEV1/ forced vital capacity (FVC)%, immunoglobulin E (IgE), serum eosinophils, and serum neutrophils between two groups; however, there was no difference in inspiratory capacity (IC) index.

**TABLE 2:** Clinical characteristics of BA patients and healthy volunteers

Index	Healthy (N = 59)	BA (N = 137)	*p*
Age	11.2 ± 3.6	12.8 ± 2.3	0.1633
Female (%)	45.76	46.72	0.8917
Allergic family history (%)	10.17	12.41	0.6167

**TABLE 3:** Clinical indicators between mild‐to‐moderate BA and severe BA

Indicator	Mild‐to‐moderate BA	Severe BA	t	*p*
FeNo (ppb)	51.21 ± 11.75	123.8 ± 12.06	35.42	<0.001
FEV1% (of predicted)	78.42 ± 11.50	64.23 ± 10.76	4.03	<0.001
FEV1/FVC (%)	84.98 ± 4.71	57.15 ± 5.32	31.98	<0.001
IC (%)	92.58 ± 23.68	90.13 ± 21.49	0.63	0.53
IgE (IU/mL)	224.68 ± 51.47	159.65 ± 67.34	6.22	<0.001
Serum eosinophils (×10^9^/L)	0.41 ± 0.23	0.53 ± 0.37	2.21	0.03
Serum neutrophils	4.10 ± 0.98	4.69 ± 1.07	3.33	0.001

Abbreviations: FEV1, forced expiratory volume in 1s; FVC, forced vital capacity; IC, inspiratory capacity; IgE, immunoglobulin E.

### 3.3 Eccentric expression of SPON2 concerning the varying degree of BA

In our pre‐experiments, we measured SPON2, OLFM4, XIST, TSIX, KDM5D, and RPS4Y1 expressions in BA patients and healthy children detected using qRT‐PCR analysis. However, there were no significant differences among OLFM4, XIST, TSIX, KDM5D, and RPS4Y1 levels between BA patients and healthy children; hence, we chose SPON2 to conduct the following experiments.

**FIGURE 2:**
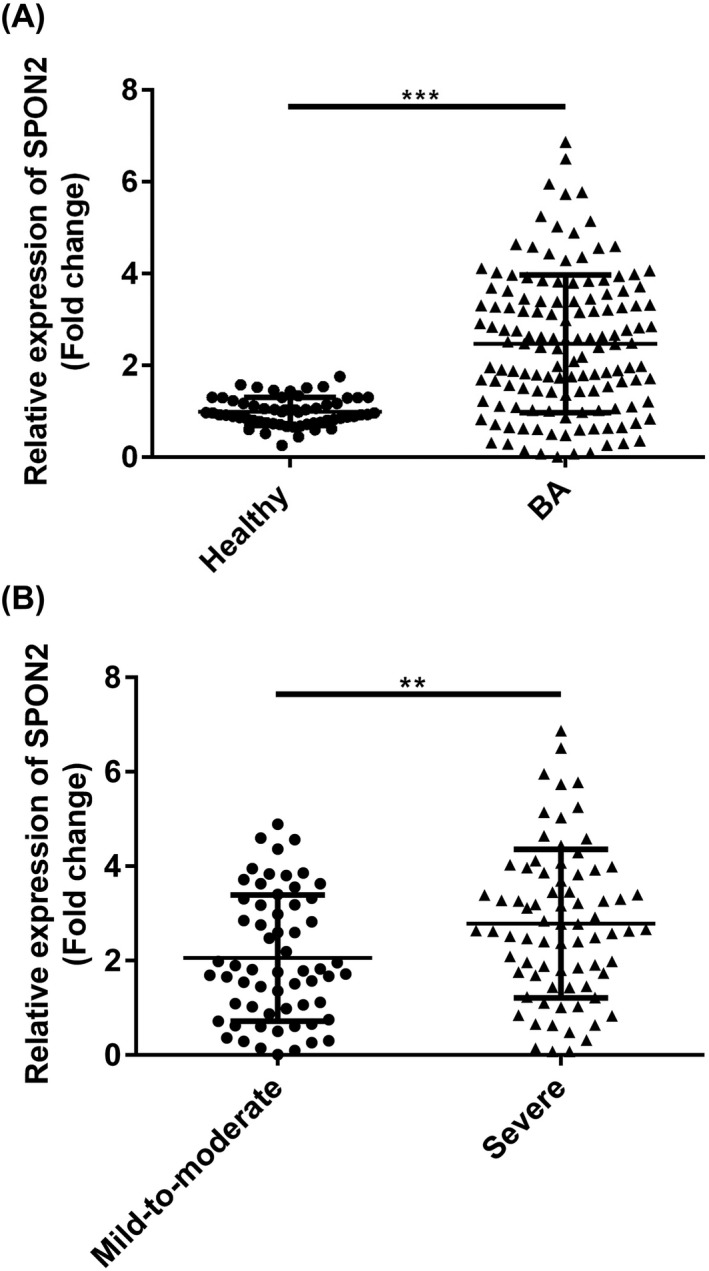
Confirmation of eccentric expressions of SPON2 in PBMCs from BA patients and healthy volunteers. (A) Levels of SPON2 were prominently increased in the healthy group. (B) Levels of SPON2 were higher in the severe group compared with the mild‐to‐moderate group. PBMC, peripheral blood mononuclear cell; SPON2, defensin alpha 4; healthy, healthy volunteers; BA, bronchial asthma

The result in Figure 2A showed that SPON2 was dramatically increased in BA patients than in healthy controls (*p* < 0.01). Moreover, through FEV1% levels, BA patients were further divided into mild‐to‐moderate and severe groups. As shown in Figure 2B, the expression of SPON2 was gradually increased with the severity of the BA (*p* < 0.01).

### 3.4 SPON2 associated with IL‐4, IL‐12, IL‐13, and IL‐17A in BA

**FIGURE 3:**
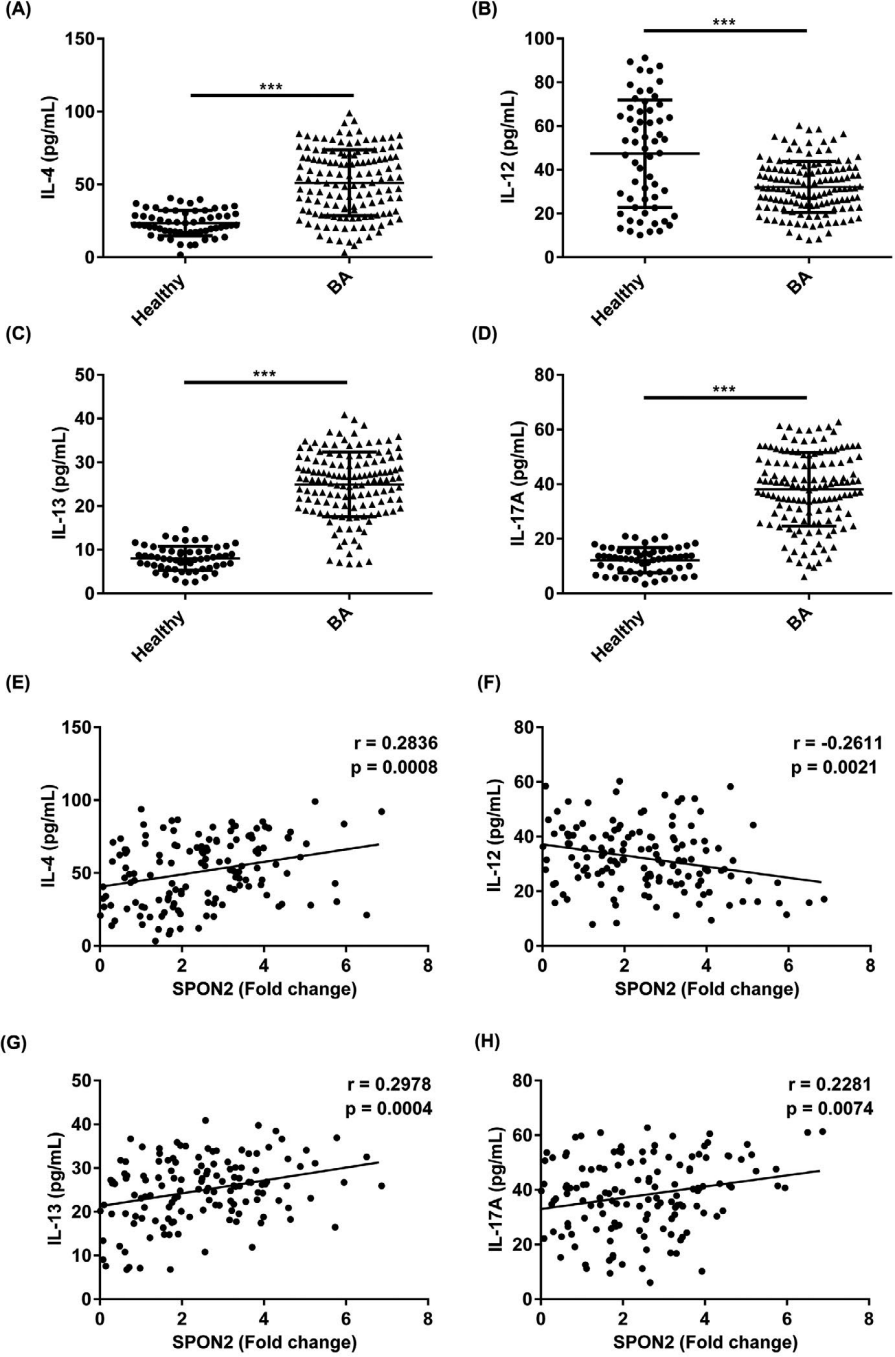
Expressions of IL‐4, IL‐12, IL‐13, and IL‐17A in BA patients and their relationship with SPON2 expression. (A‐D) Levels of IL‐4, IL‐12, IL‐13, and IL‐17A were abnormally expressed in BA patients compared with healthy controls. (E‐H) Pearson's correlation analysis measured the correlation between SPON2 and IL‐4, IL‐12, IL‐13, and IL‐17A expressions. SPON2, defensin alpha 4; healthy, healthy volunteers; BA, bronchial asthma

Using Pearson's correlation analysis, we measured correlation between SPON2 and inflammatory cytokines expressions. As shown in Figure 3, the SPON2 level was negatively correlated with IL‐12 (r = −0.2611, *p* = 0.0021). However, the SPON2 expression was significant positively correlated with IL‐4 (r = 0.2836, *p* = 0.0008), IL‐13 (r = 0.2978, *p* = 0.0004), and IL‐17A (r = 0.2281, *p* = 0.0074).

### 3.5 The diagnostic significance of SPON2 in BA

**FIGURE 4:**
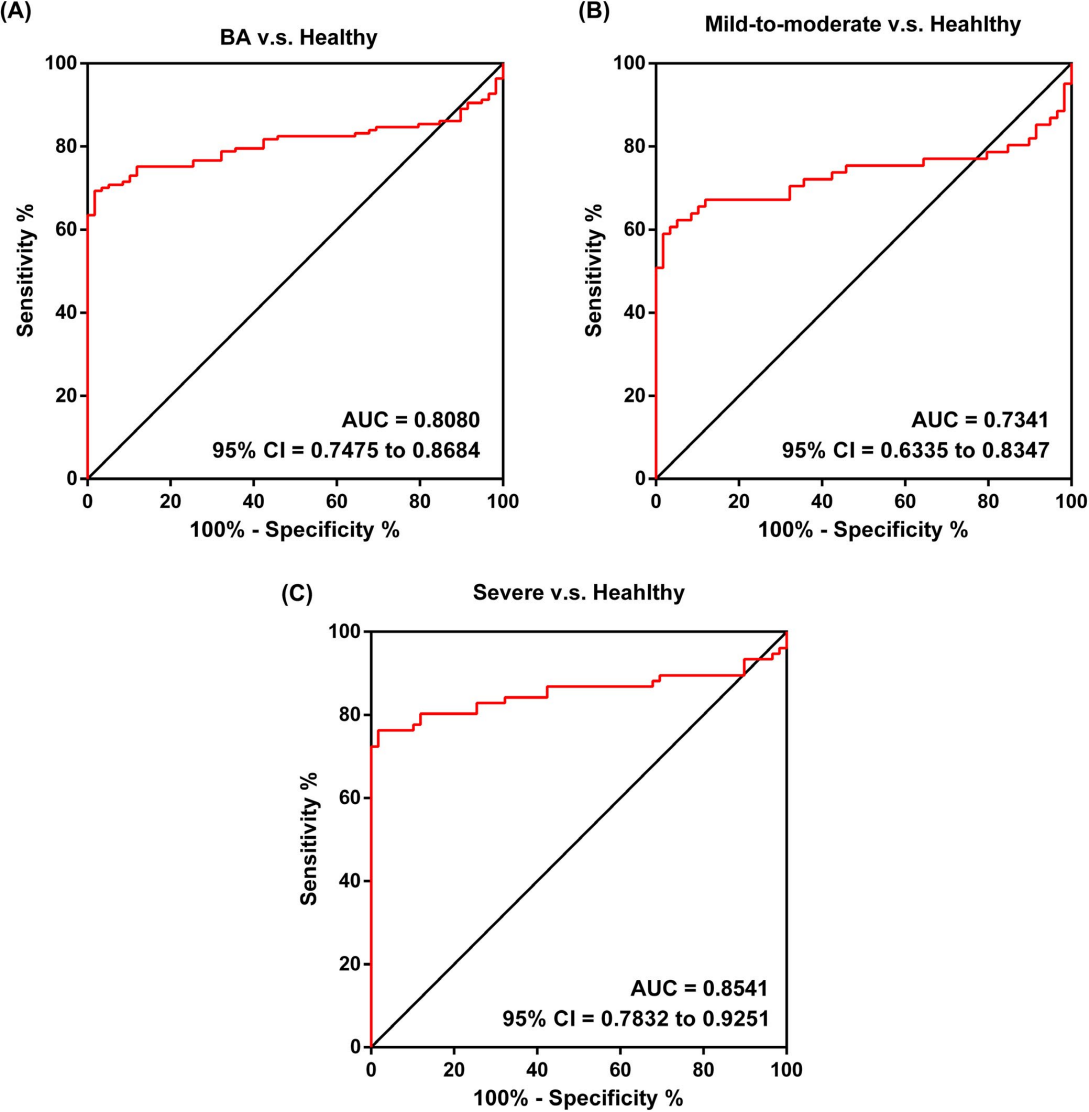
ROC analysis of SPON2 in BA. (A) The AUC of SPON2 concerning discriminating BA patients from healthy controls. (B) The AUC of SPON2 concerning mild‐to‐moderate BA patients from healthy controls. (C) The AUC of SPON2 concerning severe BA patients from healthy controls. ROC, receiver operating characteristic; AUC, area under the curve; SPON2, defensin alpha 4; healthy, healthy volunteers; BA, bronchial asthma

The ROC value of SPON2 in Figure 4A verified that SPON2 could differentiate BA patients from healthy children with an area under the curve (AUC) value of 0.8080 (95% CI = 0.7475~0.8684). Furthermore, we also conducted a ROC analysis of SPON2 to predict the severity of BA. As shown in Figure 4B,4C, the AUCs of SPON2 concerning differentiating mild‐to‐moderate BA and a severe BA from healthy controls were 0.7341 (95% CI = 0.6335~0.8347) and 0.8541 (95% CI = 0.7832~0.9251), respectively.

## 4 DISCUSSION

It was generally accepted that BA in children was caused by multiple viral and bacterial infections or exposure to specific allergens,^25^ some of which may eventually develop into persistent BA, seriously affecting children's health. Since BA's etiology and pathogenesis were still unclear, its treatment was relatively chaotic, and the abuse of hormones and antibiotics remained terrible,^26^ thereby resulting in continuous recurrences.^27^ Long‐term, high‐dose, and systemic use of hormones and antibiotics were widely applied in clinic cases, but it can also cause different degrees of systemic side effects, leading to poor compliance and unwillingness of parents to cooperate with treatment.^28^ Hence, it was of great significance to diagnose and treat BA as early as possible to arrest BA development.

BA was reported to be associated with immune system activation, addressing a pivotal role in disease progression.^29^ Previous studies verified that BA was mainly associated with IgE‐related T helper type 2 regulation,^30^ mast cells,^31^ and eosinophil recruitment.^32^ Furthermore, studies also showed the importance of B cells, T cells, macrophages, and dendritic cells in BA development.^32^, ^33^ IL‐4 was mainly produced by activated T cells^34^ and had immunomodulatory effects on B cells, T cells, mast cells, and macrophages.^35^, ^36^ Another study illustrated that IL‐4 could enhance the antigenic ability of B cells.^37^ As a growth factor secreted by T cells, IL‐4 can also maintain the Th2 cells’ proliferation,^38^ stimulating mast cell proliferation.^39^ As reported, IL‐12 was the determinant of Th1 cellular immune response,^40^ which can effectively promote the production of Th1‐related cytokines such as IFN‐γ.^41^ The role of IL‐12 concerning regulating Th1/Th2 balance contributed to BA treatment as well.^42^ Previous studies have reported that IL‐12 was significantly downregulated in BA patients than healthy.^43^ IL‐13, produced by Th2 cells, can induce B cell proliferation and IgE antibody.^44^ IL‐17A was a ligand of IL‐17, a pro‐inflammatory cytokine that can promote T cells’ activation and lead to inflammation.^45^ In serums from BA patients, IL‐13 and IL‐17A were significantly overexpressed.^46^, ^47^ Consistent with previous studies, in the present study, we found that IL‐4, IL‐13, and IL‐17A were significantly increased in BA patients and positively correlated with SPON2, whereas IL‐12 was decreased and negatively correlated with SPON2 in the BA group.

SPON2 addressed a pivotal role in various diseases, such as colorectal cancer, gastric cancer, and hepatocellular.^48^, ^49^ Loffredo et al.^18^ elucidated that SPON2 may be a vital lung morphogenetic event related to EOS. EOS was an essential airway inflammatory effector that produced inflammatory mediators, thus influencing airway hyperresponsiveness, smooth muscle thickening, and airway remodeling. The increase in EOS count was reported to be a marker of BA. In addition to EOS, FeNO, IgE, neutrophils, FEV1%, and FEV1/FVC can also be used as auxiliary indicators of BA diagnosis. The results in our study demonstrated that there were significant differences of EOS, FeNO, IgE, neutrophils, FEV1%, and FEV1/FVC between BA patients and healthy children; moreover, SPON2 expression was significantly increased in pediatric BA patients compared with healthy controls. Also, SPON2 was further increased in the severe BA group among all BA patients than in the mild‐to‐moderate group. Finally, ROC analysis confirmed the potentials of SPON2 in distinguishing BA patients from healthy controls and verifying the severity of BA.

SPON2 was remarkably increased in pediatric BA patients and correlated with the severity of illness, inflammation cytokines, and lung function indicators. Hence, SPON2 may be a feasible noninvasive biomarker contributing to BA clinical diagnosis and treatment choice.

## CONFLICT OF INTEREST

None.

## Data Availability

The datasets used and/or analyzed during the current study are available from the corresponding author on reasonable request.
